# Whole-genome sequencing of an axenic *Achromobacter* sp. strain PD1 reveals genomic potential for cyanophycin production

**DOI:** 10.1128/mra.00777-25

**Published:** 2025-08-25

**Authors:** Yingfen Zhao, Zhen Jia, Dan Li, Keith Tyo, George F. Wells

**Affiliations:** 1 Department of Civil and Environmental Engineering, Northwestern University 3270, Evanston, Illinois, USA; 2 School of Environment, Tsinghua University 538816, Beijing, China; 3 Chemical and Biological Engineering, Northwestern University 332712, Evanston, Illinois, USA; Rochester Institute of Technology, Rochester, New York, USA

**Keywords:** *Achromobacter *sp., wastewater, nitrogen recovery, biopolymer, cyanophycin

## Abstract

This study reports the whole-genome sequence of *Achromobacter* sp. strain PD1, isolated from a wastewater treatment bioreactor. Using Illumina and Nanopore sequencing, a complete genome of 6,994,232 bp was obtained. This genome contains genes predicted to encode a complete pathway for cyanophycin synthesis, a candidate for nitrogen recovery from wastewater.

## ANNOUNCEMENT

Cyanophycin, a biopolymer composed of the amino acids arginine and aspartate, offers a valuable approach to nitrogen (N) recovery from N-rich wastewater streams. Although many studies have investigated cyanophycin production in cyanobacteria (1–3), the isolation and identification of strains with cyanophycin-producing potential from wastewater treatment systems remains limited (4). In this work, we isolated the strain *Achromobacter* sp. PD1 from activated sludge and identified its genomic potential to produce cyanophycin via whole-genome sequencing.

The strain was isolated from a mixed-culture lab-scale sequencing batch reactor performing biological nitrogen and phosphorus removal from wastewater. Microbial biomass was withdrawn from the bioreactor and subjected to serial dilution prior to spread plating onto agar plates prepared using ATCC 450 T2 medium supplemented with 2% agar. Resulting colonies were further enriched in ATCC 450 T2 liquid medium at 37°C in an incubator at 150 rpm to test their ability to consume nitrogenous substrates. Promising candidates were further purified by repeated single colony streaking on Luria-Bertani (LB) agar plates, and the resulting isolate was propagated for DNA extraction and genomic sequencing.

Genomic DNA was extracted with the FastDNA SPIN kit for soil (MPBio, Santa Ana, CA, USA) according to the manufacturer’s protocol and quantified using a Qubit fluorometer (Invitrogen, San Diego, CA, USA). Both Illumina short-read sequencing and Nanopore long-read sequencing were performed at SeqCenter (Pittsburgh, PA, USA) for the isolated strain, followed by a hybrid *de novo* assembly strategy to ensure a high-quality and comprehensive whole-genome assembly.

The Illumina sequencing library was prepared using the tagmentation- and PCR-based Illumina DNA prep kit (Illumina) with custom IDT 10 bp unique dual indices and a target insert size of 280 bp (IDT, Coralville, IA, USA). The sequencing was performed on an Illumina NovaSeq X Plus sequencer, producing 2 × 151 bp paired-end reads. Adapter trimming and quality control were performed with bcl-convert (5) (v4.2.4). The Nanopore sequencing library was generated using the Oxford Nanopore Technologies (ONT) Ligation Sequencing Kit (SQK-NBD114.24). The sequencing was performed on an Oxford Nanopore MinION Mk1B sequencer, followed by super-accurate base calling and adapter trimming with Guppy (6) (v6.5.7). Residual adapters that were missed during basecalling were further removed by Porechop (7) (v0.2.4). Genome assembly was performed using Flye (8) (v2.9.2, --asm-coverage 50 --genome-size 6000000) for ONT high-quality reads. The assembled genome was further polished with Pilon (9) (v1.24) using Illumina sequencing reads to correct errors introduced by low-quality Nanopore reads. The assembly quality was evaluated using QUAST (10) (v5.2.0), and genome completeness was assessed with BUSCO (v5.4.7) based on the bacteria_odb10 lineage data set. Annotation of the assembled genome was performed using Bakta (11) (v1.8.1, db version 5.0). Default parameters were used for all software unless otherwise specified. The summary results of the sequencing, assembly, and annotation are presented in Table 1.

**TABLE 1:** Summary of sequencing, assembly, and annotation results of the sequenced genome

Sequencing parameters	Illumina	Nanopore
Number of reads	7,274,764	694,980
Size (bp)	1,075,530,433	3,910,343,322
Total bp >Q30 (20)^*a*^	982,902,161	3,551,491,299
% bp >Q30 (20)^*a*^	91	91

Genome parameters	Hybrid-assembly results	
Assembly size (bp)	6,994,232	
Contig	1	
*N*_50_ (bp)	6,994,232	
G + C content (%)	65.97%	
No. of coding genes	6,454	
Total coding sequence length (bp)	6,336,711	
Average gene length (bp)	982	
Genome coverage	702×	
Number of rRNA	13	
Number of tRNA	61	
No. of ncRNAs	7	

^*a*^ Total bp >Q20* for Nanopore sequencing results.

**Fig 1:**
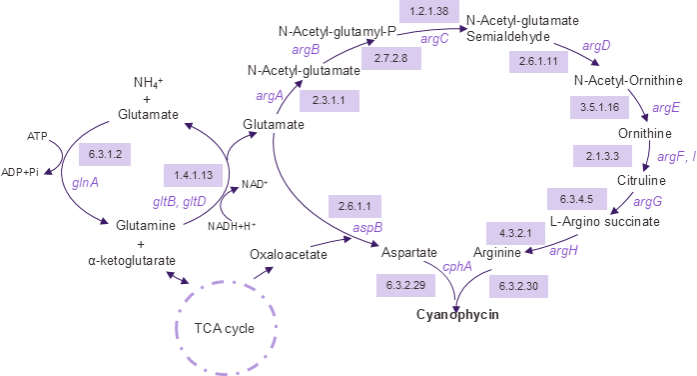
Cyanophycin synthesis pathway of *Achromobacter* sp. PD1 reconstructed from whole-genome sequencing results. Enzyme Commission (EC) numbers in purple boxes represent the specific enzymes catalyzing each step in the pathway. Gene names (in purple text) correspond to the encoding genes for these enzymes.

Whole-genome sequencing results were analyzed using the genome analysis tool--METABOLIC to explore the potential metabolic pathways of *Achromobacter* sp. PD1 (12). METABOLIC-G pipeline intaking individual genome sequence was used to obtain KEGG ortholog (KO) identifiers with KO IDs and the corresponding gene hits (12). The KO identifiers were subsequently mapped to metabolic pathways using the KEGG Mapper tool (13), which allowed reconstruction of the cyanophycin synthesis pathway (Fig. 1).

## Data Availability

The genome data of *Achromobacter* sp. strain PD1 have been deposited in NCBI GenBank under BioProject accession number PRJNA1221536. The genome accession number is CP183349. The version described in this paper is the first version. Raw sequence reads have been submitted to the NCBI Sequence Read Archive under Bioproject number PRJNA1221325. The accession numbers are SRR32280757 for Illumina sequencing reads and SRR32280756 for Nanopore sequencing reads.
